# Supplementary material for: A UMLS-based spell checker for natural language processing in vaccine safety
Source: BMC Med Inform Decis Mak. 2007 Feb 12;7:3. doi: 10.1186/1472-6947-7-3 (PMC1805499; doi:10.1186/1472-6947-7-3)
Supplement: Additional file 13 — RAP application directory. Sets up directory for RAP (RDF application for PHP) source code [file 1472-6947-7-3-S13.gz › rap/doc/tests/Test_Set_Operations.htm]

Test Set Operations


### RDF API for PHP V0.9.1

# Test: Set Operations

This example is part of the RAP - Rdf API for PHP documentation.

  

### 1. Generate and show two models

`define("RDFAPI_INCLUDE_DIR", "./../api/");  
include(RDFAPI_INCLUDE_DIR . "RDFAPI.php");`

`echo "<h3>1. Generate and show two MemModels</h3>";`

`// Create empty MemModel  
$model = ModelFactory::getDefaultModel();` `$model->setbaseURI("http://www.bizer.de");`

`$model2 = ModelFactory::getDefaultModel();  
$model2->setbaseURI("http://www.bizer.de/zwei");`

`// Create Nodes and add statements to models  
$myhomepage = new Resource("http://www.bizer.de/welcome.html");  
$creator = new Resource("http://purl.org/dc/elements/1.1/creator");  
$me = new Resource("mailto:chris@bizer.de");  
$model->add(new Statement($myhomepage, $creator, $me));  
$model2->add(new Statement($myhomepage, $creator, $me));`

`$creation_date = new Resource("http://www.example.org/terms/creation-date");  
$August16 = new Literal("August 16, 2002");  
$model->add(new Statement($myhomepage, $creation_date, $August16));  
$model2->add(new Statement($myhomepage, $creation_date, $August16));`

`$language = new Resource("http://www.example.org/terms/language");  
$deutsch = new Literal("Deutsch", "de");  
$model->add(new Statement($myhomepage, $language, $deutsch));`

`$name = new Resource("http://www.example.org/terms/Name");  
$chrisbizer = new Literal("Chris Bizer");  
$model2->add(new Statement($me, $name, $chrisbizer));`

`// Output as Table  
echo "<h5>Model 1</h5>";  
$model->writeAsHtmlTable();  
echo "<h5>Model 2</h5>";  
$model2->writeAsHtmlTable();`

**Output:**

**Model 1**

|  |  |  |  |
| --- | --- | --- | --- |
| **Base URI:** http://www.bizer.de# | | | **Size:** 3 |
| **No.** | **Subject** | **Predicate** | **Object** |
| 1. | Resource: http://www.bizer.de/welcome.ht ml | Resource: http://purl.org/dc/elements/1. 1/creator | Resource: mailto:chris@bizer.de |
| 2. | Resource: http://www.bizer.de/welcome.ht ml | Resource: http://www.example.org/terms/c reation-date | Literal: August 16, 2002 |
| 3. | Resource: http://www.bizer.de/welcome.ht ml | Resource: http://www.example.org/terms/l anguage | Literal: Deutsch **(xml:lang="de")** |

  
**Model 2**

|  |  |  |  |
| --- | --- | --- | --- |
| **Base URI:** http://www.bizer.de/zwei# | | | **Size:** 3 |
| **No.** | **Subject** | **Predicate** | **Object** |
| 1. | Resource: http://www.bizer.de/welcome.ht ml | Resource: http://purl.org/dc/elements/1. 1/creator | Resource: mailto:chris@bizer.de |
| 2. | Resource: http://www.bizer.de/welcome.ht ml | Resource: http://www.example.org/terms/c reation-date | Literal: August 16, 2002 |
| 3. | Resource: mailto:chris@bizer.de | Resource: http://www.example.org/terms/N ame | Literal: Chris Bizer |

  
  
  

### 2. Make some tests

`echo "<h2>2. Make some tests</h2>";  
echo "Test: Model 2 contains any statements from model 1 :".$model2->containsAny($model)."<p>";
  
echo "Test: Model 1 contains any statements from model 2 :".$model->containsAny($model2)."<p>";
  
echo "Test: Model 2 contains all statements from model 1 :".$model2->containsAll($model)."<p>";
  
echo "Test: Model 1 contains all statements from model 2 :".$model->containsAll($model2)."<p>";
  
echo "Test: Model 1 equals model 2 :".$model->equals($model2)."<p>";`

**Output:**

Test: Model 2 contains any statements from model 1 :1

Test: Model 1 contains any statements from model 2 :1

Test: Model 2 contains all statements from model 1 :

Test: Model 1 contains all statements from model 2 :

Test: Model 1 equals model 2 :

### 3. Unite model 1 and model 2

`echo "<h3>3. Unite model 1 and model 2</h3>";  
$model3 =& $model->unite($model2);  
$model3->writeAsHtmlTable();`

**Output:**

|  |  |  |  |
| --- | --- | --- | --- |
| **Base URI:** http://www.bizer.de# | | | **Size:** 4 |
| **No.** | **Subject** | **Predicate** | **Object** |
| 1. | Resource: http://www.bizer.de/welcome.ht ml | Resource: http://purl.org/dc/elements/1. 1/creator | Resource: mailto:chris@bizer.de |
| 2. | Resource: http://www.bizer.de/welcome.ht ml | Resource: http://www.example.org/terms/c reation-date | Literal: August 16, 2002 |
| 3. | Resource: http://www.bizer.de/welcome.ht ml | Resource: http://www.example.org/terms/l anguage | Literal: Deutsch **(xml:lang="de")** |
| 4. | Resource: mailto:chris@bizer.de | Resource: http://www.example.org/terms/N ame | Literal: Chris Bizer |

### 4. Intersect model 1 and model 2

`echo "<h3>4. Intersect model 1 and model 2</h3>";  
$model4 =& $model->intersect($model2);  
$model4->writeAsHtmlTable();`

**Output:**

|  |  |  |  |
| --- | --- | --- | --- |
| **Base URI:** http://www.bizer.de# | | | **Size:** 2 |
| **No.** | **Subject** | **Predicate** | **Object** |
| 1. | Resource: http://www.bizer.de/welcome.ht ml | Resource: http://purl.org/dc/elements/1. 1/creator | Resource: mailto:chris@bizer.de |
| 2. | Resource: http://www.bizer.de/welcome.ht ml | Resource: http://www.example.org/terms/c reation-date | Literal: August 16, 2002 |

### 5. Substract model 2 from model 1

`echo "<h3>5. Substract model 2 from model 1</h3>";  
$model5 =& $model->subtract($model2);  
$model5->writeAsHtmlTable();`

**Output:**

|  |  |  |  |
| --- | --- | --- | --- |
| **Base URI:** http://www.bizer.de# | | | **Size:** 1 |
| **No.** | **Subject** | **Predicate** | **Object** |
| 1. | Resource: http://www.bizer.de/welcome.ht ml | Resource: http://www.example.org/terms/l anguage | Literal: Deutsch **(xml:lang="de")** |

### 6. Reify model 1

`echo "<h3>6. Reify model 1</h3>";  
$model6 =& $model->reify();  
$model6->writeAsHtmlTable();`

**Output:**

|  |  |  |  |
| --- | --- | --- | --- |
| **Base URI:** http://www.bizer.de# | | | **Size:** 12 |
| **No.** | **Subject** | **Predicate** | **Object** |
| 1. | Blank Node: bNode1 | RDF NS Node: http://www.w3.org/1999/02/2 2-rdf-syntax-ns#type | RDF NS Node: http://www.w3.org/1999/02/2 2-rdf-syntax-ns#Statement |
| 2. | Blank Node: bNode1 | RDF NS Node: http://www.w3.org/1999/02/2 2-rdf-syntax-ns#subject | Resource: http://www.bizer.de/welcome.ht ml |
| 3. | Blank Node: bNode1 | RDF NS Node: http://www.w3.org/1999/02/2 2-rdf-syntax-ns#predicate | Resource: http://purl.org/dc/elements/1. 1/creator |
| 4. | Blank Node: bNode1 | RDF NS Node: http://www.w3.org/1999/02/2 2-rdf-syntax-ns#object | Resource: mailto:chris@bizer.de |
| 5. | Blank Node: bNode2 | RDF NS Node: http://www.w3.org/1999/02/2 2-rdf-syntax-ns#type | RDF NS Node: http://www.w3.org/1999/02/2 2-rdf-syntax-ns#Statement |
| 6. | Blank Node: bNode2 | RDF NS Node: http://www.w3.org/1999/02/2 2-rdf-syntax-ns#subject | Resource: http://www.bizer.de/welcome.ht ml |
| 7. | Blank Node: bNode2 | RDF NS Node: http://www.w3.org/1999/02/2 2-rdf-syntax-ns#predicate | Resource: http://www.example.org/terms/c reation-date |
| 8. | Blank Node: bNode2 | RDF NS Node: http://www.w3.org/1999/02/2 2-rdf-syntax-ns#object | Literal: August 16, 2002 |
| 9. | Blank Node: bNode3 | RDF NS Node: http://www.w3.org/1999/02/2 2-rdf-syntax-ns#type | RDF NS Node: http://www.w3.org/1999/02/2 2-rdf-syntax-ns#Statement |
| 10. | Blank Node: bNode3 | RDF NS Node: http://www.w3.org/1999/02/2 2-rdf-syntax-ns#subject | Resource: http://www.bizer.de/welcome.ht ml |
| 11. | Blank Node: bNode3 | RDF NS Node: http://www.w3.org/1999/02/2 2-rdf-syntax-ns#predicate | Resource: http://www.example.org/terms/l anguage |
| 12. | Blank Node: bNode3 | RDF NS Node: http://www.w3.org/1999/02/2 2-rdf-syntax-ns#object | Literal: Deutsch **(xml:lang="de")** |
